# Mental health in medical, dentistry and veterinary students: cross-sectional online survey

**DOI:** 10.1192/bjo.2018.61

**Published:** 2018-10-25

**Authors:** Duleeka Knipe, Chloe Maughan, John Gilbert, David Dymock, Paul Moran, David Gunnell

**Affiliations:** Vice Chancellor's Research Fellow, Population Health Sciences, Bristol Medical School, University of Bristol, UK; Strategic Projects Manager, Students’ Union, University of Bristol, UK; Medical Student, Faculty of Health Sciences, University of Bristol, UK; Professor of Dental Education, Bristol Dental School, University of Bristol, UK; Professor of Psychiatry, Population Health Sciences, Bristol Medical School, University of Bristol and National Institute for Health Research Bristol Biomedical Research Centre, University Hospitals Bristol, National Health Science Foundation Trust and University of Bristol, UK; Professor of Epidemiology, Population Health Sciences, Bristol Medical School, University of Bristol and National Institute for Health Research Bristol Biomedical Research Centre, University Hospitals Bristol, National Health Science Foundation Trust and University of Bristol, UK

**Keywords:** Anxiety disorders, alcohol disorders, self-harm, depressive disorders, student

## Abstract

**Background:**

The mental health of university students, especially medical students, is of growing concern in the UK.

**Aim:**

To estimate the prevalence of mental disorder in health sciences students and investigate help-seeking behaviour.

**Method:**

An online survey from one English university (*n* = 1139; 53% response rate) collected data on depression (using the nine-item Patient Health Questionnaire), anxiety (seven-item Generalised Anxiety Disorder Assessment), alcohol use (Alcohol Use Disorders Identification Test), self-harm and well-being, as well as help seeking.

**Results:**

A quarter of the students reported symptoms of moderate/severe depression and 27% reported symptoms of moderate/severe anxiety. Only 21% of students with symptoms of severe depression had sought professional help; the main reason for not seeking help was fear of documentation on academic records.

**Conclusions:**

The study highlights the extent of mental health problems faced by health science students. Barriers to help seeking due to concerns about fitness-to-practise procedures urgently need to be addressed to ensure that this population of students can access help in a timely fashion.

**Declaration of interest:**

None.

## Background

There is growing concern about the mental health of students in higher education; it is estimated that one in five students worldwide have a mental disorder.^1^ Particular attention needs to be given to students on medical, dentistry and veterinary courses as they may experience more stress than students on other courses, given the longer course duration (and therefore overall higher tuition fees), the longer hours spent in study and exposure to potentially distressing clinical conditions.^2^ They may also have certain personality characteristics (e.g. perfectionism) that predispose them to a higher risk of developing mental illness during their studies.^3^ There is also evidence that once they leave university, healthcare professionals – particularly females – may be at increased risk of suicide and mental health problems.^4^^,^^5^ Therefore, it is important that they develop healthy responses for managing any mental health difficulties during their undergraduate years.

About one in four medical students experience depression/depressive symptoms,^6^ but recent evidence from the UK is lacking as the last prevalence estimate was assessed over 8 years ago.^7^ Understanding the prevalence of mental health disorders in health science students is important given the unique characteristics of these courses, which may include clinical placements some distance away from the traditional university support systems, and therefore might mean that help-seeking practices are different from those of other students. Health science students may face additional barriers to accessing help amidst concerns over General Medical Council^8^ and General Dental Council^9^ fitness-to-practise regulations. Most previous studies have focused on the prevalence of, and risk factors for, depression in health science students.^6^^,^^10^ Few studies have investigated their help-seeking behaviours and barriers to support utilisation.^11^

## Aims of the study

Using data from an online survey of health science students at a UK university, we aimed to address the following research questions:

- What is the prevalence of depression, anxiety, alcohol-use disorders, self-harm and poor mental well-being in students of health sciences courses?
- What proportion of those with severe depression and persistent suicidal thoughts access professional support services?
- Among those students with severe depression and persistent suicidal thoughts, what are the reported barriers to accessing help?

## Method

### Outline of the study

Altogether, 2133 undergraduate students studying medicine (1140 students), dentistry (344) or veterinary sciences (649) at an English university were invited to take part in an anonymised online survey (May 2017). Students were sent several reminders over a 2-week survey period.

Data on age, gender and course type were collected along with data on: (a) depression, using the nine-item Patient Health Questionnaire (PHQ-9);^12^ (b) anxiety, using the seven-item Generalised Anxiety Disorder Assessment (GAD-7);^13^ (c) self-harm (with and without suicidal intent); (iv) alcohol use disorders, using the Alcohol Use Disorders Identification Test (AUDIT); and (v) well-being, using the Warwick–Edinburgh Mental Wellbeing Scale (WEMWBS).^14^ Although PHQ-9 and GAD-7 are not diagnostic tools, they have both been used to estimate the prevalence of depression^12^ and anxiety^13^ in the general population. We used item 9 in the PHQ-9 to identify students experiencing suicidal thoughts: ‘Over the last two weeks, have you been bothered by thoughts that you would be better off dead, or of hurting yourself in some way?’ Students were asked about their self-harm behaviour in the past 12 months using two questions: (a) ‘In the past 12 months, have you ever hurt yourself on purpose in any way (e.g. by taking an overdose of pills or by cutting yourself)?’ and (b) ‘On those occasions in the past 12 months when you have hurt yourself on purpose, have you ever seriously wanted to kill yourself?’ These questions were used to categorise students into those who have self-harmed with or without suicidal intent. Students were also asked questions on the sources of help used in the 2 weeks prior to the survey. Help-seeking options included reaching out to a partner, friend, parent, other relative, general practitioner (GP), member of the faculty of health sciences staff, member of university support staff, someone else not listed above and using the internet. Students were also asked to list reasons for not seeking help by selecting one or more of the following response items: (a) I have not had a problem, (b) lack of time, (c) lack of confidentiality, (d) concern that ‘no one will understand my problems’, (e) I did not know where to find help, (f) stigma of mental healthcare, (g) fear of unwanted intervention, (h) fear of documentation on academic record, (i) difficulty with access to care, (j) lack of available services and (k) other (please specify).

To help contextualise the quantitative findings, participants were asked the following free-text response question: ‘Please list up to three things you feel could be done to improve emotional and mental health support for students at your institution.’

### Data analysis

Stata release 15 (Windows) was used for all analyses. A complete case analysis was conducted, excluding students with missing data for any of the variables included in this analysis (97 (8%) students had missing data). We used the recommended cut-offs for each of the validated scales.^15^^–^^17^ Students were categorised as having moderate to severe (PHQ-9/GAD-7 score of 10 or more) or severe (PHQ-9 score of 20 or more; GAD-7 score of 15 or more) anxiety or depression. On the original PHQ-9 questionnaire, the penultimate question is ‘Over the last 2 weeks, how often have you been bothered by moving or speaking so slowly that other people could have noticed? Or the opposite – being so fidgety or restless that you have been moving around a lot more than usual?’ In this survey, the question was split into a question relating to slow movement/speech and a question relating to fidgetiness or restless. For scoring purposes, we used the highest score obtained for either of these questions. For the AUDIT scores, we used a cut-off of 8 or more to indicate hazardous drinking, and a score of 16 or more to indicate harmful drinking. We also categorised the WEMWBS score for each student as being above or below the national average of 23.63 (combined average for both genders).^17^ We used logistic regression to assess whether course of study was associated with risk of poor mental health outcomes, adjusted for age and gender.

The main analysis included all students, regardless of course or year of study. The timing of the survey coincided with an exam period for some students, and exam-related stress may have influenced levels of anxiety. We therefore reviewed the exam timetables and conducted a sensitivity analysis, stratifying on the basis of whether survey respondents were undertaking exams at the time of the survey, to explore how examination participation influences the main findings. Dental students were excluded from this analysis as all years of this course were sitting or about to sit exams. Year 1 medical and year 4 veterinary students had exams around the survey period. We compared these students with the cohort on either side of this year of study with the largest number of students to maximise power. We also present results by year of study.

The free-text responses were subjected to a thematic qualitative analysis.

Ethical approval was obtained through the Faculty of Health Sciences at the University of Bristol (no. 49861). Participants gave informed consent before taking part in the study.

## Results

### Quantitative analysis

The response rate from the 2133 students invited to participate in the study was as follows: 51% of medical students, 65% of dentistry students and 51% of veterinary science students. A total of 1139/2133 students were included in the analysis (53% of eligible students): 51% were medical, 20% were dentistry and 29% were veterinary science students. The median age of participants was 21 years and 76% were female, with higher response rates in females (64%) than males (44%).

Roughly a quarter of students reported symptoms of moderate to severe depression (male 19%; female 27%) and 27% had moderate to severe anxiety (male 20%; female 30%) (Table 1). Nearly 40% of students were categorised as engaging in hazardous drinking behaviour. One percent of students reported persistent (daily over 2 weeks) suicidal thoughts (16% reported any suicidal thoughts); 7% reported self-harming in the past year, with 2% self-harming with suicidal intent.

**Table 1.** Prevalence of depression symptoms, anxiety symptoms, alcohol-use disorders, self-harm and poor well-being scores in health science students

	Total *n* (%)	Course *n* (%)			Odds ratio^a^		
		Medicine	Dentistry	Veterinary sciences	Medicine	Dentistry	Veterinary sciences
*n*	1139	583	223	333			
Median age (IQI)	21 (20, 23)	21 (20, 23)	21 (20, 22)	21 (20, 23)			
Gender							
Male	269 (23.6)	171 (29.3)	52 (23.3)	46 (13.8)			
Female	870 (76.4)	412 (70.7)	171 (76.7)	287 (86.2)			
Depression (PHQ-9 score)							
≥10	287 (25.2)	130 (22.3)	79 (35.4)	78 (23.4)	1.0	1.9 (1.3, 2.6)	1.0 (0.7, 1.4)
≥20	24 (2.1)	13 (2.2)	7 (3.1)	4 (1.2)	1.0	1.4 (0.5, 3.5)	0.5 (0.2, 1.5)
Anxiety (GAD-7 score)							
≥10	311 (27.3)	145 (24.9)	87 (39.0)	79 (23.7)	1.0	1.9 (1.4, 2.6)	0.9 (0.6, 1.2)
≥15	137 (12.0)	69 (11.8)	39 (17.5)	29 (8.7)	1.0	1.6 (1.0, 2.4)	0.7 (0.4, 1.0)
Alcohol-use disorders (AUDIT score)							
≥8	425 (37.3)	219 (37.6)	85 (38.1)	121 (36.3)	1.0	1.0 (0.8, 1.4)	1.1 (0.8, 1.4)
≥16	58 (5.1)	32 (5.5)	12 (5.4)	14 (4.2)	1.0	1.0 (0.5, 2.0)	0.9 (0.5, 1.8)
Self-harm/suicidal behaviour							
Suicidal thoughts^b^	11 (1.0)	6 (1.0)	4 (1.8)	1 (0.3)	1.0	1.7 (0.5, 6.3)	0.3 (0, 2.6)
Self-harm (regardless of intent)^c^	83 (7.3)	41 (7.0)	17 (7.6)	25 (7.5)	1.0	1.0 (0.6, 1.9)	1.0 (0.6, 1.7)
Self-harm with intent^c^	25 (2.2)	13 (2.2)	5 (2.2)	7 (2.1)	1.0	1.0 (0.3, 2.8)	1.0 (0.4, 2.5)
Well-being (WEMWBS score)							
Below national average^d^	795 (69.8)	370 (63.5)	179 (80.3)	246 (73.9)	1.0	2.3 (1.6, 3.3)	1.5 (1.1, 2.0)

IQI, interquartile interval; PHQ-9, nine-item Patient Health Questionnaire; GAD-7, seven-item Generalised Anxiety Disorder Assessment; AUDIT, Alcohol Use Disorders Identification Test; WEMWBS, Warwick–Edinburgh Mental Wellbeing Scale.

a. Compared with medical students, adjusted for age and gender.

b. Nearly every day in the past 2 weeks; 16.2% reported suicidal thoughts several days or more (medical: 15.6%; dentistry: 22.0%; veterinary science: 13.2%).

c. In the past 12 months.

d. Below the national average of 23.63.

**Fig. 1:**
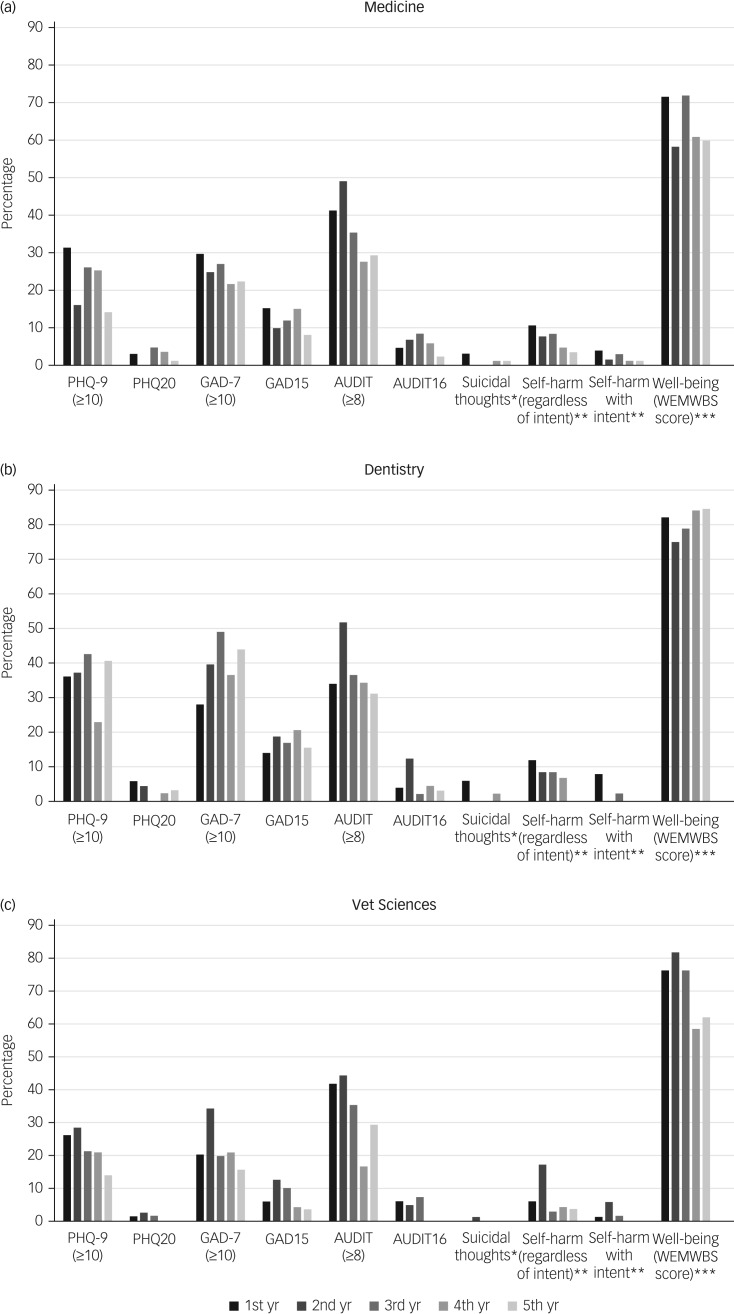
Prevalence of depression symptoms, anxiety symptoms, alcohol-use disorders, self-harm and poor well-being scores in health science students by year of study and course. *Nearly every day in the past 2 weeks. **In the past 12 months. ***Below the national average.

A large proportion (70%) of students reported lower than average well-being scores. There was evidence that a higher proportion of dentistry students (compared with medical students) had moderate depression, higher levels of anxiety and lower well-being scores. The veterinary students did not report higher levels of depression and anxiety symptoms; however, they did report having lower well-being scores compared with medical students. The prevalence of poor mental health appeared to be higher in the first few years of study and declined as students remained in education among medical and veterinary science students, but this pattern was not apparent in dental students (Fig. 1).

Of those students who reported symptoms of severe depression, only 21% accessed any professional help, and of those who had frequent suicidal thoughts, 27% accessed help (Table 2). The most common form of professional help sought was via the student's GP. A quarter of students with symptoms of severe depression sought help from the internet.

**Table 2.** Help seeking in students with severe depression symptoms (PHQ9 ≥20) or persistent suicidal thoughts

	PHQ-9 score ≥20 *n* (%)	Suicidal thoughts^a^ *n* (%)
Accessed any help	24 (100)	11 (100)
Accessed professional help^b^	5 (20.8)	3 (27.3)
Which sources of help have you used in the past 2 weeks?^c^		
Partner	10 (41.7)	7 (63.6)
Friend	15 (62.5)	8 (72.7)
Parent	12 (50.0)	5 (45.5)
Other relative/family member	3 (12.5)	2 (18.2)
General practitioner	5 (20.8)	3 (27.3)
Faculty staff	0 (0)	0 (0)
University support staff	1 (4.2)	2 (18.2)
Other	3 (12.5)	2 (18.2)
Internet	6 (25.0)	4 (36.4)
None of the above	0 (0)	0 (0)

PHQ-9, nine-item Patient Health Questionnaire.

a. Nearly every day in the past 2 weeks.

b. Professional help meaning general practitioner, faculty staff or university support staff. Each student is counted once, but could select more than one option.

c. Students could select more than one option and therefore students may be counted more than once.

Students with severe depression symptoms (and who did not seek help) reported fear of documentation (50%), lack of time (46%) and fear of unwanted intervention (46%) as the most common barriers to seeking help (Table 3). Few students (17%) reported that they did not know where to find help or that they felt there was a lack of available services. Of those students with persistent suicidal thoughts, similar concerns were raised as for those students with severe depression, but in addition, concerns over lack of confidentiality (46%) and stigma (55%) were highlighted. Comparison of barriers by degree course indicated that fear of documentation was the most commonly reported barrier in medical students (69%), whereas lack of time was the most commonly reported barrier to help seeking among dentistry students (57%). There were too few (*n* = 4) responses from veterinary students with severe depression symptoms to draw out any patterns.

**Table 3.** Reported barriers for help seeking in students with severe depression symptoms or persistent suicidal thoughts

	PHQ-9 score ≥20 *n* (%)				Suicidal thoughts^a^ *n* (%)			
	Overall	Medicine	Dentistry	Veterinary sciences	Overall	Medicine	Dentistry	Veterinary sciences
*n*	24	13	7	4	11	6	4	1
Main barriers for help-seeking^b^								
I have not had a problem	1 (4.2)	0 (0)	1 (14.3)	0 (0)	0 (0)	0 (0)	0 (0)	0 (0)
Lack of time	11 (45.8)	7 (53.8)	4 (57.1)	0 (0)	6 (54.5)	3 (50.0)	3 (75.0)	0 (0)
Lack of confidentiality	8 (33.3)	6 (46.2)	1 (14.3)	1 (25.0)	5 (45.5)	4 (66.7)	1 (25.0)	0 (0)
Concern that no one will understand my problems	7 (29.2)	6 (46.2)	1 (14.3)	0 (0)	4 (36.4)	2 (33.3)	2 (50.0)	0 (0)
I did not know where to find help	4 (16.7)	2 (15.4)	2 (28.6)	0 (0)	4 (36.4)	2 (33.3)	2 (50.0)	0 (0)
Stigma of mental healthcare	6 (25.0)	5 (38.5)	0 (0)	1 (25.0)	6 (54.5)	4 (66.7)	1 (25.0)	1 (100)
Fear of unwanted intervention	11 (45.8)	6 (46.2)	3 (42.9)	2 (50.0)	7 (63.6)	4 (66.7)	3 (75.0)	0 (0)
Fear of documentation on academic record	12 (50.0)	9 (69.2)	1 (14.3)	2 (50.0)	7 (63.6)	4 (66.7)	2 (50.0)	1 (100)
Difficulty with access to care	4 (16.7)	3 (23.1)	0 (0)	1 (25.0)	0 (0)	0 (0)	0 (0)	0 (0)
Lack of available services	4 (16.7)	2 (15.4)	0 (0)	2 (50.0)	2 (18.2)	1 (16.7)	0 (0)	1 (100)
Other	5 (20.8)	3 (23.1)	1 (14.3)	1 (25.0)	3 (27.3)	2 (33.3)	1 (25.0)	0 (0)

a. Nearly every day in the past 2 weeks.

b. Students could indicate more than one barrier.

The sensitivity analysis revealed that medical students who were sitting exams in year 1 had lower depression scores than students in year 2 who were not sitting their exams (Supplementary table 1 available at https://doi.org/10.1192/bjo.2018.61) at the time of the survey. However, there were no other differences between students who were either sitting or just about to sit exams during the survey period compared with students not taking exams.

### Free-text responses on how to improve provision of mental health services

The free-text responses from 20 students who scored more than 20 on PHQ-9 were subject to thematic analysis. The emerging themes related to the need to improve access to support, improve staff training and culture, and concerns relating to fitness-to-practise policies and procedures. To some extent, these issues mirror the results from the quantitative analysis regarding the most common barriers to accessing help.

Concerns about access to support typically related to issues of lack of time. Some students noted a need to access out-of-hours GP services or counselling due to the clash of opening hours with teaching and clinical placement hours, as well as the fact that they sometimes had clinical placements many miles away from the university campus.

Free-text comments relating to fitness to practise highlighted students' concerns about how mental health difficulties would be documented on their academic records or used by staff. For example, one participant noted ‘you really need to address and constantly remind students that problems will not go on their academic records’. This finding was supported by a secondary thematic analysis of the responses from all students, regardless of PHQ-9 score.

## Discussion

Our results confirm previous reports of high levels of mental health problems among medical, dentistry and veterinary students.^6^^,^^18^^,^^19^ Relatively few health science students with severe depression had sought professional help, with the most frequently reported reason being fear of documentation on their academic record, a concern that was particularly prevalent among medical students. This is despite the fact that over 80% of students with high depression (PHQ-9) scores were aware of the available sources of help and reported thinking that these were accessible. Contrary to reports of high levels of alcohol misuse among university students,^20^ the levels of harmful or hazardous drinking in students in this study did not differ from those in the similarly aged UK population.^21^^,^^22^

The current survey has several strengths. Validated questionnaires were used and our response rate was similar to those reported in other recent medical student surveys.^23^^,^^24^ Furthermore, we were able to compare the prevalence of mental health problems across three undergraduate health professional degree courses. We also explored the use of professional help sources and identified potential barriers for help seeking. Importantly, to the best of our knowledge, this is the first UK-based survey that has investigated barriers to help seeking for mental health problems within the medical student population.

However, these findings need to be interpreted in light of certain limitations. The survey was carried out at a single UK institution and the findings may not be generalisable to other student populations. The timing of the survey, during an exam period for some students (particularly dentistry students), may have influenced responses, leading to an overestimation of the frequency of symptoms generally experienced by students. However, findings from the sensitivity analysis were broadly similar to those obtained from the main analysis. Given the snapshot, cross-sectional nature of the study, we are unable to determine what proportion of students were experiencing sustained symptoms. Finally, although the response rate in this survey is similar to that of other surveys of this nature, the findings are likely to have been affected by non-response bias given the higher response rate among females and their higher prevalence of mental illness. Furthermore, if the individuals who took part in the survey differed from those who did not (e.g. if those with depression were more or less likely to respond) then this will influence our prevalence estimates. Unfortunately we are unable to explore this in detail due to the anonymity of the survey.

A systematic review which included 195 studies investigating the prevalence of depression in medical students found a somewhat lower prevalence of depression symptoms (18.3%, 95% CI 12.8–25.4%, PHQ-9 ≥10) to the rate found in this study (22.3%).^6^ The majority of studies included in the review tended to have more male participants (average 44%) than in the current study (29%), which may explain the slightly higher prevalence observed. This gender difference is in keeping with most population-based studies.^25^ The prevalence estimate of moderate/severe depression for dentistry and veterinary students is in line with previous research.^18^^,^^19^ Levels of anxiety were higher in dentistry and veterinary science courses compared with previous studies.^18^^,^^19^ These prevalence differences might be because the previous studies did not use validated scales to assess anxiety or because they were conducted in different cultural settings. The prevalence of anxiety symptoms in medical students was similar to previous studies.^10^ In our study, the prevalence of suicidal ideation within the past 2 weeks (PHQ-9 question) in medical students was significantly higher (15.6%) than the pooled estimate of the two previous studies (conducted in China and Germany) that measured suicidal ideation in this way (7.4%, 95% CI 5.9–9.2%).

Compared with previous studies,^26^ we found higher than expected levels of suicidal ideation among dentistry students, as well as higher odds of depression and anxiety in dentistry students compared with medical students. To the best of our knowledge, this is the first head-to-head comparison between health science students. The qualitative elements of the survey indicated that this may relate to issues around workload and assessment that were particular to the dentistry course; some student cohorts were undergoing a continual programme of assessment, with more regular examinations than their medical peers. We are also aware that the mental health of dentistry students was a topic that was being given considerable attention among both staff and students, and this may underlie the high response rate among dentistry students.

There have been few studies that have investigated barriers for help seeking.^27^^,^^28^ An important finding of this study is that fear of documentation, which might affect professional students’ fitness to practise, has been reported as a barrier for help seeking, particularly for medical students. The exploration of the free-text fields of this survey indicates that there may be misconceptions within the student population around how disclosure of mental illness will be documented and whether it will affect their ability to meet fitness-to-practise requirements. Similar concerns over academic reprisal have been noted in another study in medical students in the US.^29^ Evidence from a UK-based study suggests that medical students are more likely to self-manage or seek informal help through family/friends who are also in the medical profession, and that this behaviour is learned early on in a student's career.^30^ This preference for treating personal medical problems may also be partly driven by fear of institutional documentation.

The findings of this study highlight the importance of providing appropriate pathways to help and treatment for health science students. This is particularly important as they enter the workforce and face the additional demands of professional/clinical practice. Further qualitative research and randomised controlled trials will help identify which interventions could be effective in preventing and supporting students with mental illness.

Given their vulnerability to mental health problems, health faculty students should – like the general population – be regularly reminded of the health benefits derived from regular exercise, a healthy diet, enough sleep and the avoidance of harmful alcohol consumption. In addition, mindfulness has increasingly been shown to protect against stress^31^ and may therefore offer particular benefits to health science students. Finally, from the outset of their training, health science students should be provided with much clearer information about fitness-to-practise procedures. The timely provision of this information may reduce the fear associated with seeking help and may encourage distressed students to seek help more promptly.^32^

## Supplementary material

For supplementary material accompanying this paper visit http://dx.doi.org/10.1192/bjo.2018.61. click here to view supplementary material
