# Supplementary material for: Mental health in medical, dentistry and veterinary students: cross-sectional online survey
Source: BJPsych Open. 2018 Oct 25;4(6):441–6. doi: 10.1192/bjo.2018.61 (PMC6235980; doi:10.1192/bjo.2018.61)
Supplement: Supplementary file 1 [file S2056472418000613sup001.docx]

**Supplementary Table 1** – **Comparison of medical and vet students with and without exams during the survey period**

|  |  | Medicine | | Vet Sciences | |
| --- | --- | --- | --- | --- | --- |
|  |  | Year 1 - Exam | Year 2 - No Exam | Year 4 - Exam | Year 3 - No Exam |
| Depression (PHQ-9 score) | |  |  |  |  |
|  | ≥10 | 21 (15.9) | 41 (31.3) | 15 (21.1) | 5 (20.8) |
|  | ≥20 | 0 (0) | 4 (3.1) | 1 (1.4) | 0 (0) |
| Anxiety (GAD-7 score) | |  |  |  |  |
|  | ≥10 | 33 (25) | 39 (29.8) | 14 (19.7) | 5 (20.8) |
|  | ≥15 | 13 (9.8) | 20 (15.3) | 7 (9.9) | 1 (4.2) |
| Alcohol use disorders (AUDIT score) | |  |  |  |  |
|  | ≥8 | 65 (49.2) | 54 (41.2) | 25 (35.2) | 4 (16.7) |
|  | ≥16 | 9 (6.8) | 6 (4.6) | 5 (7) | 0 (0) |
| Self-harm/Suicidal behaviour | |  |  |  |  |
|  | Suicidal thoughts^a^ | 0 (0) | 4 (3.1) | 0 (0) | 0 (0) |
|  | Self-harm (regardless of intent)^b^ | 10 (7.6) | 14 (10.7) | 2 (2.8) | 1 (4.2) |
|  | Self-harm with intent^b^ | 2 (1.5) | 5 (3.8) | 1 (1.4) | 0 (0) |
| Wellbeing (WEMWBS score) | |  |  |  |  |
|  | Below national average^c^ | 77 (58.3) | 94 (71.8) | 54 (76.1) | 14 (58.3) |

^a^ nearly everyday in the last 2 weeks

^b^ in the last 12 months

^c^ Below the national average = 23.63 (1)
